# Characterizing white matter connectome abnormalities in patients with temporal lobe epilepsy using threshold‐free network‐based statistics

**DOI:** 10.1002/brb3.3643

**Published:** 2024-08-05

**Authors:** Daniel Y Chu, Theodore P Imhoff‐Smith, Veena A Nair, Timothy Choi, Anusha Adluru, Camille Garcia‐Ramos, Kevin Dabbs, Jedidiah Mathis, Andrew S Nencka, Lisa Conant, Jeffrey R Binder, Mary E Meyerand, Andrew L Alexander, Aaron F Struck, Bruce Hermann, Vivek Prabhakaran, Nagesh Adluru

**Affiliations:** ^1^ Department of Radiology University of Wisconsin School of Medicine and Public Health Madison Wisconsin USA; ^2^ Department of Neurology University of Wisconsin School of Medicine and Public Health Madison Wisconsin USA; ^3^ Department of Medical Physics University of Wisconsin Madison Madison Wisconsin USA; ^4^ Department of Neurology Medical College of Wisconsin Milwaukee Wisconsin USA; ^5^ Department of Radiology Medical College of Wisconsin Milwaukee Wisconsin USA; ^6^ Waisman Center University of Wisconsin Madison Madison Wisconsin USA; ^7^ Department of Neurology William S. Middleton Veterans Hospital Madison Wisconsin USA; ^8^ Department of Psychiatry University of Wisconsin School of Medicine and Public Health Madison Wisconsin USA

**Keywords:** cognitive impairment, connectome, diffusion‐weighted imaging, generalized tonic–clonic seizures, temporal lobe epilepsy

## Abstract

**Introduction:**

Emerging evidence illustrates that temporal lobe epilepsy (TLE) involves network disruptions represented by hyperexcitability and other seizure‐related neural plasticity. However, these associations are not well‐characterized. Our study characterizes the whole brain white matter connectome abnormalities in TLE patients compared to healthy controls (HCs) from the prospective Epilepsy Connectome Project study. Furthermore, we assessed whether aberrant white matter connections are differentially related to cognitive impairment and a history of focal‐to‐bilateral tonic–clonic (FBTC) seizures.

**Methods:**

Multi‐shell connectome MRI data were preprocessed using the DESIGNER guidelines. The IIT Destrieux gray matter atlas was used to derive the 162 × 162 structural connectivity matrices (SCMs) using MRTrix3. ComBat data harmonization was applied to harmonize the SCMs from pre‐ and post‐scanner upgrade acquisitions. Threshold‐free network‐based statistics were used for statistical analysis of the harmonized SCMs. Cognitive impairment status and FBTC seizure status were then correlated with these findings.

**Results:**

We employed connectome measurements from 142 subjects, including 92 patients with TLE (36 males, mean age = 40.1 ± 11.7 years) and 50 HCs (25 males, mean age = 32.6 ± 10.2 years). Our analysis revealed overall significant decreases in cross‐sectional area (CSA) of the white matter tract in TLE group compared to controls, indicating decreased white matter tract integrity and connectivity abnormalities in addition to apparent differences in graph theoretic measures of connectivity and network‐based statistics. Focal and generalized cognitive impaired TLE patients showcased higher trend‐level abnormalities in the white matter connectome via decreased CSA than those with no cognitive impairment. Patients with a positive FBTC seizure history also showed trend‐level findings of association via decreased CSA.

**Conclusions:**

Widespread global aberrant white matter connectome changes were observed in TLE patients and characterized by seizure history and cognitive impairment, laying a foundation for future studies to expand on and validate the novel biomarkers and further elucidate TLE's impact on brain plasticity.

## Graphical abstract

This study investigates multi‐shell diffusion‐weighted white matter connectome abnormalities in patients with temporal lobe epilepsy. Threshold‐free network‐based statistics of the harmonized structural connectivity matrices reveal significant widespread decreases in cross‐sectional area of the white matter connectome, demonstrating decreased white matter integrity throughout the whole brain. Correlation of findings with patient cognitive impairment statuses and focal‐to‐bilateral tonic–clonic seizures reveal modest associations with aberrant white matter structural connectome.

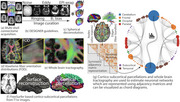

## LIMITATIONS

Although our results indicated some significant differences between the control and TLE group, the sample size was modest for the FBTC and cognitively impaired TLE patient analyses and a future study with a greater number of participants would be beneficial to elaborate and further validate the findings from this work. To further establish the robustness of these findings across different analysis parameters, future studies should, for example, explore other network parcellations with higher spatial resolution atlases, such as the Brainnetome Atlas. In addition, although previous work showed that lateralization of TLE may have implications for structural and functional networks (Lee et al., 2018; Whelan et al., 2018), the modest sample size inhibited our ability to test the hemispheric heterogeneity of our findings across left, right, and bilateral TLE. The ECP, which aims to understand phenotypical heterogeneity in the more general population of TLE, contains a more representative sample of TLE patients than typically investigated in surgical series, and thus, only a subset of participants (35%) underwent the gold standard of ictal monitoring. Future studies may be able to scrutinize the laterality effects in a completely ictally monitored cohort, but such cases may capture mostly the severe end of the epilepsy spectrum which would be less representative of the general population of TLE. One pervasive problem in the study of TLE is the underlying heterogeneity of the disease and the lack of a definitive gold standard for diagnosis. Even with the best tools available, most patients with bitemporal lobe epilepsy require 41.6 days of EEG monitoring to detect such bilateral seizures, and some may require up to 1 year (King‐Stephens et al., 2015). Thus, we can never be sure of a “definitive” seizure laterality and TLE diagnosis. Additionally, drastic limiting of participant selection results in its own type of selection bias where results may be questionably extrapolated to the representative and less complicated TLE patient. The purpose of the ECP was to get a cross‐sectional sampling of patients with TLE defined in the typically clinical and pragmatic method with the help and oversight of epileptologists.

## 1 INTRODUCTION

Epilepsy is the 4th most common neurological disorder, with a prevalence of over 3.4 million in the USA and more than 46 million worldwide (Zack & Kobau, 2017; GBD 2016 Neurology Collaborators, 2019). In adults, temporal lobe epilepsy (TLE) is the most common form of focal epilepsy (Téllez‐Zenteno & Hernández‐Ronquillo, 2012). Although some TLE patients undergo long‐term remission, lifelong chronic TLE is debilitating and heavily associated with several cognitive, somatic, and psychiatric comorbidities (Anderson et al., 2011). Predicting which patients will respond favorably to surgery or anti‐seizure medication is imperative for the optimization of treatment and planning; however, current methods for these predictions are inadequate. Currently, there are no reliable biomarkers for accurate assessment of treatment efficacy, disease progression, or the severity of neurological and psychiatric consequences in TLE patients. Previous literature with modest sample sizes reported variable results for MRI biomarkers, which expose a fundamental lack of understanding in the pathophysiological processes underlying epilepsy progression and remission (Arfanakis et al., 2002; Dow et al., 2004; Janecek et al., 2013; Zahn et al., 2009).

Emerging evidence has demonstrated that TLE involves a series of networks characterized by pathologies, such as hyperexcitability and other seizure‐related neural plasticity (Maccotta et al., 2013; Richardson, 2012; Stefan & Lopes da Silva, 2013; Terry et al., 2012). Recent Diffusion Tensor Imaging (DTI) studies have reported widespread microstructural abnormalities in subcortical white matter related to reduced neurite density (Winston et al., 2020) as well as microstructural abnormalities in the corpus callosum, cingulum, and external capsule (Hatton et al., 2020) of TLE patients. Other studies have explored the interplay of focal seizure areas, such as the hippocampus, with its connectome‐level effects using diffusion MRI connectomics, revealing significant remodeling of connectome topology and structurally governed functional dynamics in TLE (Bernhardt et al., 2019). In contrast to DTI studies, diffusion‐weighted imaging (DWI) connectome studies map the entirety of white matter connections across the whole brain, providing a comprehensive overview of how different brain regions are structurally connected. Few studies have characterized whole brain white matter connectome abnormalities in TLE patients compared to controls in a large‐scale patient population.

The Epilepsy Connectome Project (ECP) is a large‐scale dataset that includes robust cognitive assessments with neuropsychological batteries and connectivity measurements of patients with sporadic TLE (Struck et al., 2021; Hwang et al., 2019; Garcia‐Ramos et al., 2023). In the present study, we utilized the ECP multi‐shell connectome diffusion‐weighted MRI (ms‐dMRI) data to evaluate structural connections and dynamic network interactions and used graph theoretical descriptive analysis of topology to characterize the white matter connectome in TLE patients. We further tested white matter connectome associations with cognitive impairment status and focal‐to‐bilateral tonic–clonic (FBTC) seizure history (previously known as generalized tonic–clonic seizures) in TLE patients. FBTC seizures begin in a focal area and spread across the whole brain bilaterally, making this clinical characteristic a natural variable to correlate with the whole‐brain networks measured by ms‐dMRI. Most participants from this sample at the date of their study visit had not had an FBTC seizure within the past year, and most that did were between 20 and 30 years old. Thus, we chose to focus on lifetime occurrence. We hypothesize that TLE patients will reveal discrete white matter connectivity abnormalities reflected by differences in graph theoretic measures of connectivity and network‐based statistics compared to healthy controls (HCs). In addition, we test whether these abnormalities correlate with both lifetime occurrence of FBTC and cognitive impairment status as characterized by earlier work with the ECP (Hermann et al., 2020). These analyses elucidate whole‐brain differences in white matter networks of TLE patients, lay a foundation for the use of ms‐dMRI‐based structural connectivity measures in developing novel biomarkers of TLE, and provide further insights into our understanding of TLE as a brain network disorder involving ongoing seizure‐induced neural plasticity.

## 2 METHODS

### 2.1 Participants

Participants are from the ECP, a multisite collaborative research project between the University of Wisconsin Madison and the Medical College of Wisconsin (MCW) conducted between 2016 and 2018. This prospective study was approved by the institutional review board at the MCW, and all participants provided written informed consent.

The inclusion criteria for TLE patients required two of the following: (1) clinical semiology consistent with seizures of temporal lobe origin, (2) previous MRI consistent with mesial temporal sclerosis (MTS) or hippocampal atrophy, (3) electroencephalography (EEG) revealing temporal intermittent rhythmic delta or epileptiform activity over the temporal lobe(s), and (4) EEG monitoring video evidence of temporal lobe onset seizure. Diagnosis of TLE was overseen by epileptologists, and potential cases were reviewed by the clinicians scrutinizing the previously mentioned clinical evidence (structural brain MRI, interictal EEG, clinical semiology, and ictal‐EEG monitoring for those who were admitted to epilepsy monitoring units), all available past and current medical records surrounding seizure care, management, and diagnosis. Contentious diagnoses were reviewed and discussed by all epileptologists of the study to reach a consensus. Patients were excluded if they had lesions other than MTS, active infectious, autoimmune, or inflammatory seizure etiology. Controls were healthy adults, and exclusion criteria included history of learning disability, substance abuse, brain injury, psychiatric illnesses, and current vasoactive medication use. All participants had no MRI contraindications.

The majority of studies utilizing the ECP participants have investigated the resting‐state functional connectivity and cortical surface morphometry, and only one study explored the DWI by utilizing machine learning tools to determine intrinsic TLE structural connectome phenotypes (Struck et al., 2021; Hwang et al., 2019; Garcia‐Ramos et al., 2023).

### 2.2 Data acquisition

MRI images were acquired on 3T GE MR750 scanners (General Electric) using a Nova 32‐channel head coil. T1‐weighted structural images were acquired using a 3D gradient‐echo pulse (MPRAGE) sequence (repetition time (TR) = 604 ms, echo time (TE) = 2.516 ms, inversion time = 1060 ms, flip angle = 8°, field of view (FOV) = 25.6 cm, and 0.8 mm isotropic). T2‐weighted structural images were acquired using a 3D fast spin‐echo (CUBE) sequence (TR = 2500 ms, TE = 94.398 ms, flip angle = 90°, FOV = 25.6 cm, and 0.8 mm isotropic). Diffusion‐weighted MRI (dMRI) data were acquired using the high‐angular resolution diffusion imaging approach (Tuch et al., 2003). The ECP dMRI acquisition uses multiband Echo Planar Imaging (EPI) (Moeller et al., 2010) with slide acceleration factor 3 that includes a total of 4 runs, with 2 runs with 75‐directions each, and opposite phase encoding polarity (AP and PA), and 2 runs with 76 directions each. The DWI images had the additional acquisition parameters: alternating *b* = 1000 s/mm^2^ and *b* = 2000 s/mm^2^, and 9 *b* = 0 volumes.

### 2.3 Data processing and statistical analyses

**FIGURE 1:**
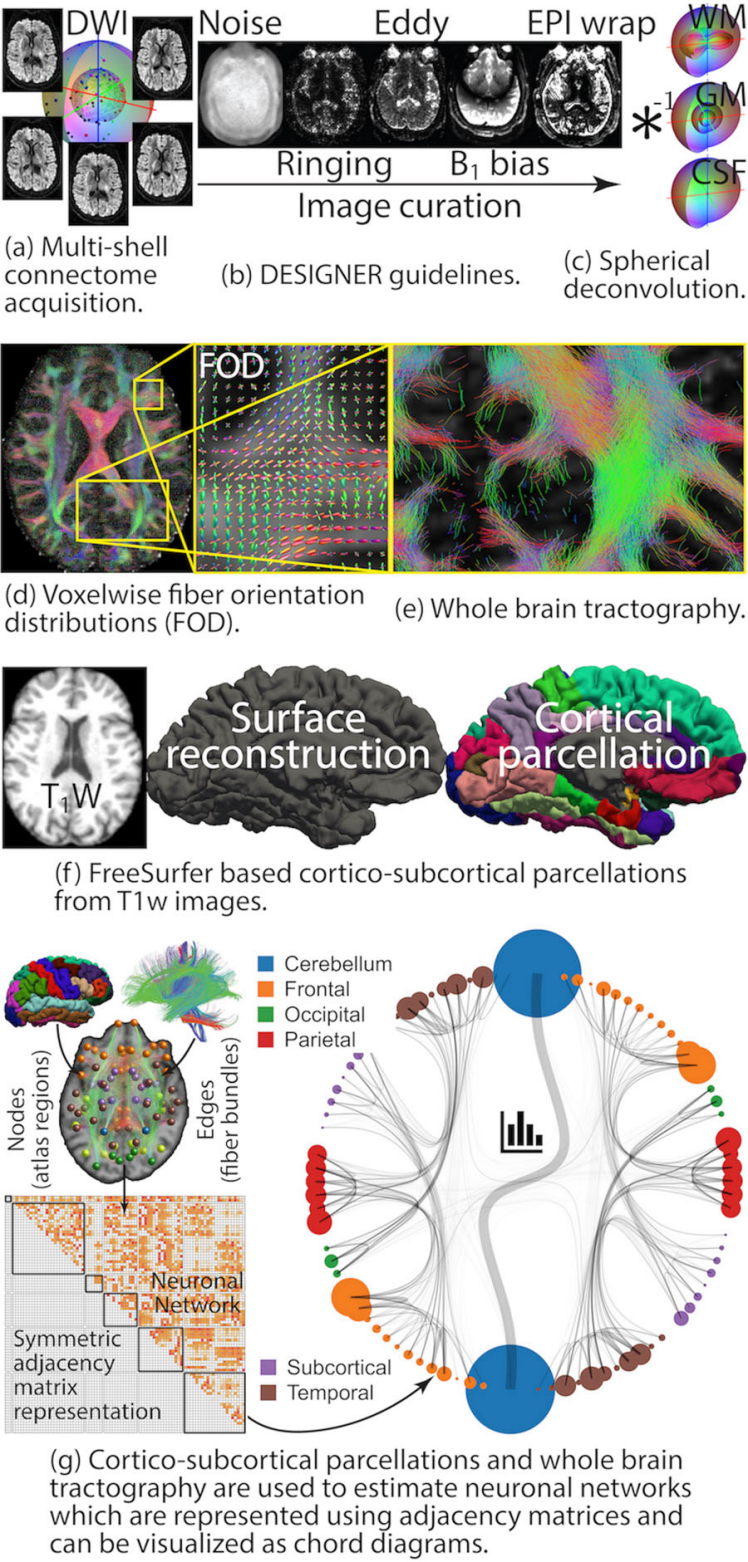
Overview of the connectography reconstruction from connectome quality multi‐shell diffusion‐weighted imaging (DWI) data: (a–c) preprocessing of the multi‐shell data followed DESIGNER guidelines (Ades‐Aron et al., 2018) which included the removal or mitigation of artifacts such as noise, Gibb's ringing, distortion due to eddy currents, B_1_‐bias and EPI wrapping due to field inhomogeneities using tools in FMRIB Software Library (FSL) (Jenkinson et al., 2012), Advanced Normalization Tools (ANTs) (Avants et al., 2009), and MRTrix3 (Tournier et al., 2019); (d and e) the curated DWI data were deconvolved using constrained optimization with the shell and major tissue‐specific response functions to estimate fiber orientation distributions (FOD) at each voxel in the brain; (f) anatomical cortico‐subcortical gray matter atlas regions identified using FreeSurfer reconstruction and parcellation of the gray matter used to define the nodes in the neuronal networks; (g) underlying white matter fiber bundles connecting pairs of these nodes were used to define the edges. These networks can be represented using symmetric matrices, which encode the adjacency or connectedness of the nodes.

Modern tools from the emerging field of network neuroscience were used to quantify and assess structural network connectivity pathways. We quantified the organization of structural connections as edges, which previous network neuroscience research has shown to provide insights into network communication efficiency patterns (Bullmore & Sporns, 2009; Sotiropoulos et al., 2013) and used threshold‐free network‐based statistics (TFNBS), robust statistical network analysis technique, for predictive modeling (Baggio et al., 2018). Figure 1 provides an overview of the connectography reconstruction from connectome quality multi‐shell DWI data. Preprocessing of the data followed DESIGNER guidelines, which included the removal or mitigation of artifacts such as noise, Gibb's ringing, distortion due to eddy currents, B_1_‐bias, and EPI warping due to field inhomogeneities. The curated DWI data were deconvolved using constrained optimization with the shell and major tissue‐specific response functions to estimate fiber orientation distributions (FOD) at each voxel in the brain. Anatomical cortico‐subcortical gray matter atlas regions were identified using the IIT Destrieux atlas in FreeSurfer. The Destrieux atlas is a well‐validated atlas that provides a good balance between computational complexity for generating reliable tractography and regional resolution. The identified gray matter boundaries were used to define the nodes in the neuronal networks. Cross‐sectional areas (CSA) of the underlying white matter fiber bundles connecting pairs of these nodes were used to define the edges of the connectivity networks. The resulting networks were represented using 162 × 162 symmetric matrices, which encode the adjacency or connectedness of the nodes. The tractography was performed using the tckgen command in MRtrix3. Fifteen million streamlines were generated by dynamic seeding in the whole brain using the FODs and a probabilistic algorithm (iFOD2) which performs second‐order integration over FODs. The step size was set to the default value of ½ the voxel size. The tract stopping criteria were also set to the defaults of 45° angular threshold for curving, minimum length of twice the voxel size, and maximum length of 100 times the voxel size. The FOD amplitude cutoff was set to 0.05. The tcksift2 algorithm from MRtrix3 was used to filter and compute the weights for each streamline used in computing the final edge weights in the networks. Fiber density estimates were heuristically shrunk based on the presence of gray matter using the fd_scale_gm option for tcksift2. SIFT proportionality constant 𝜇 was also saved which is used to scale the network adjacency matrices. These networks can be represented using symmetric matrices, which encode the adjacency or connectedness of the nodes.

Parametric data harmonization of the connectivity matrices was employed using neuroComBat (Fortin et al., 2017) to account for the DV25–DV26 change, a major software of the GE MR scanner. This included a change from sagittal to axial acquisition. Harmonization addressed the batch/protocol effects by accounting for additive (on the bias) and multiplicative (on the variance) effects of the scanner software and slice direction in acquisition on connectivity outcomes using a linear model by preserving group, age, and sex effects. These correction effects are the expected values of the empirically estimated Bayesian prior distributions either parametrically via Expectation‐Maximization or non‐parametrically via importance sampling. Such a prior‐based approach is robust when there are small sample sizes per each protocol. Figure S1 illustrates the effects of harmonization on the adjacency matrices (edge weights) before permutation testing.

**FIGURE 2:**
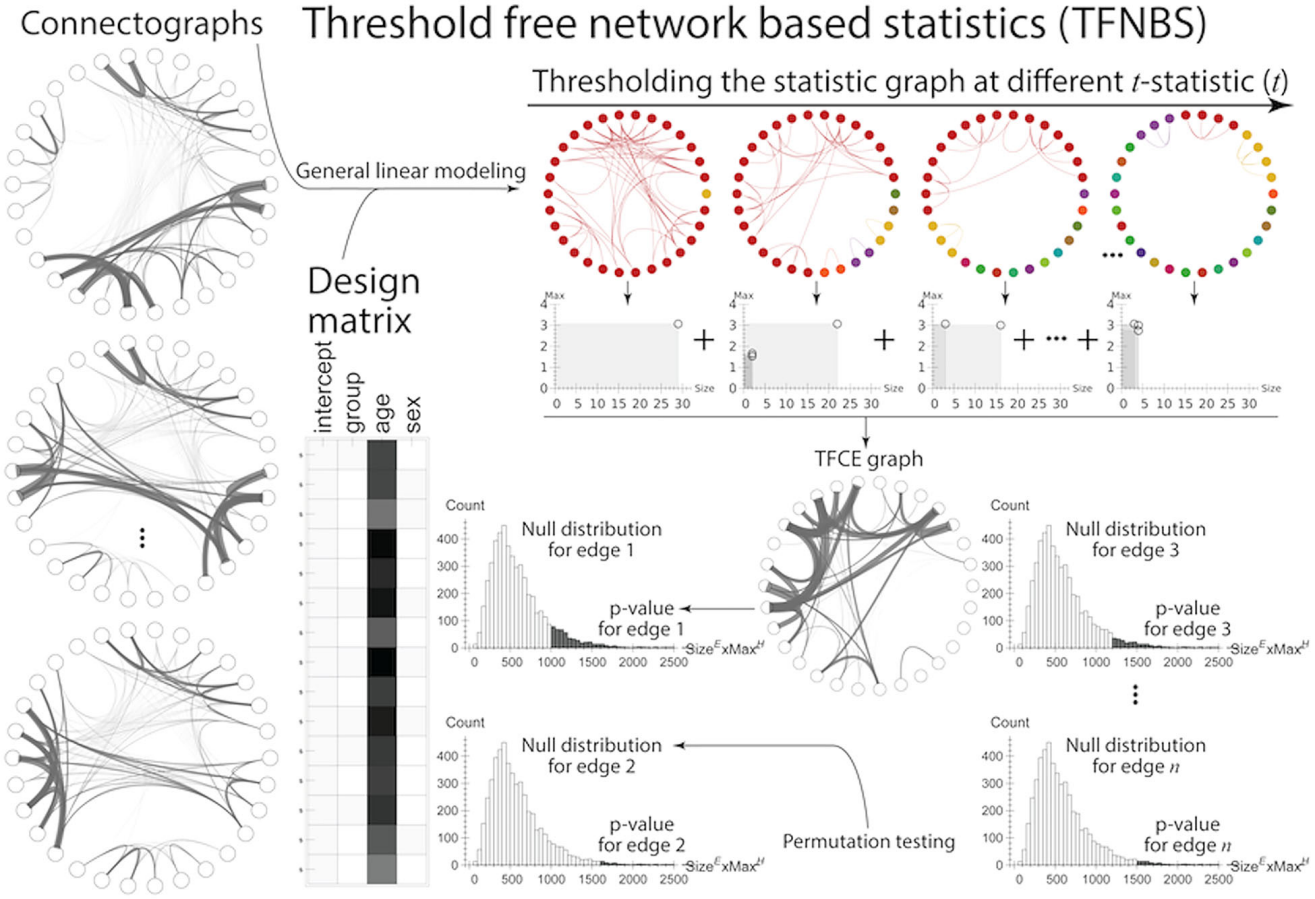
Overview of **t**hreshold‐free network‐based statistics (TFNBS). General linear modeling is used to get statistic graphs from connectographs. The statistic graphs are thresholded at different thresholds to cluster the nodes. The product of size and max of the clusters are summed across the thresholds to obtain threshold‐free cluster‐enhanced statistic graph. The null distribution can be obtained for each connection or maximum across the connections using permutation testing. Comparing each connection in the unthresholded statistic graph with (1) max‐null or (2) connection‐specific null results in family wise error‐corrected or uncorrected *p*‐value graphs, respectively.

The resulting connectographs were fed into the TFNBS that are described in Figure 2. General linear modeling (GLM) generated statistic graphs from connectographs, which were thresholded to cluster the nodes (Baggio et al., 2018). The product of the size and maximum of the clusters were summed across the thresholds to obtain threshold‐free cluster‐enhanced statistic graphs. Permutation testing with 5000 permutations was used to obtain null distributions for each connection or the maximum across the connections. Comparing each connection in the unthresholded statistic graph with (1) max‐null or (2) connection‐specific null results in family‐wise error‐corrected or uncorrected *p*‐value graphs, respectively. Statistically, significant results at *p* ≤ .05 were reported. Age and sex were used as nuisance variables in the GLM. Results were then correlated with cognitive impairment status and patient history of FBTC seizures.

## 3 RESULTS

### 3.1 Demographics of study participants

Multi‐shell DWI connectome data were collected from 142 participants, including 92 TLE patients (36 males, mean age = 40.1 ± 11.7 years) and 50 HCs (25 males, mean age = 32.6 ± 10.2 years) ranging from ages 18–60. The participant sociodemographic and clinical characteristics are shown in Table 1. The average years of education of the TLE patient group and HC group are 14.6 ± 2.8 and 16.1 ± 2.7 years, respectively. The full‐scale intelligence quotient average score for the TLE group is 101.7 ± 13.8 and 113.4 ± 15.1 for HCs. In the TLE group, 62 patients have a positive FBTC seizure history, 21 patients have never experienced an FBTC seizure, and the remaining patients had an unknown FBTC history. Notable clinical characteristics of the TLE patients include 12.2 ± 20.3 mean lifetime FBTC seizures, mean age of seizure onset of 22.4 ± 14.2, mean disease duration of 18.0 ± 14.0, mean seizure frequency of 5.8 ± 10.6 per month, and mean number of anti‐seizure medications of 1.77 ± 0.97. The seizure lateralization and diagnosis of TLE were determined by board‐certified neurologists, in accordance with criteria defined by the International League Against Epilepsy, based on a matrix of interictal scalp EEG or intracranial video‐EEG telemetry, seizure semiology, and neuroimaging evaluation. Forty‐eight TLE patients have left lateralized seizures, 22 right seizure lateralization, 10 bilateral, and 12 are unknown. In terms of cognitive impairment, 9 TLE patients (12%) had generalized cognitive impairment, 25 patients (33.3%) had focal cognitive impairment, and 41 patients (54.7%) had no cognitive impairment. The remaining patients had unknown cognitive impairment statuses. From the list of eligible ECP participants, 32 participants were excluded due to missing DWI data, poor quality control data, missing participant variables, or low number of links on harmonization matrices.

**TABLE 1:** Participant demographics.

	TLE patients (*n* = 92)	Controls (HCs) (*n* = 50)
Age (mean ± SD)*	40.1 ± 11.7	32.6 ± 10.2
**Sex (M/F)**	36/56	25/25
Education (mean ± SD)*	14.6 ± 2.8	16.1 ± 2.7
FSIQ (mean ± SD)*	101.7 ± 13.8	113.4 ± 15.1
**FBTC (±)**	62/21	N/A
**Number of lifetime FBTC (mean ± SD)**	12.2 ± 20.3	N/A
**Age of seizure onset (mean ± SD)**	22.4 ± 14.2	N/A
**Disease duration (mean ± SD)**	18.0 ± 14.0	N/A
**Seizure frequency per month (mean ± SD)**	5.8 ± 10.6	N/A
**No. of ASMs**	1.77 ± 0.97	N/A
**Seizure lateralization (L/R/B/U)**	45/19/10/18	N/A
**Generalized cognitive impairment (*n*; %)**	9; 12%	47
**Focal cognitive impairment (*n*; %)**	25; 33.3%	47
**No cognitive impairment (*n*; %)**	41; 54.7%	47

Abbreviations: ASMs, anti‐seizure medications; B, bilateral; FBTC, focal‐to‐bilateral tonic–clonic; FSIQ, full‐scale intelligence quotient; HC, healthy controls; L, left; R, right; SD, standard deviation; TLE, temporal lobe epilepsy; U, unknown.

*Significantly different between TLE and HCs (*p* < .05).

### 3.2 Group differences between TLE and HC

**FIGURE 3:**
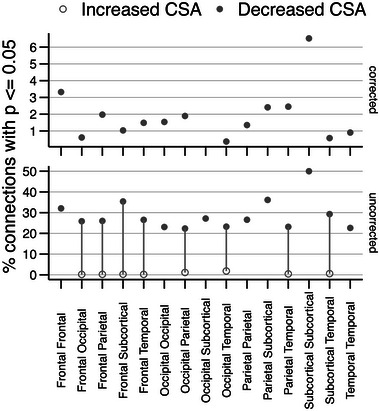
Summary findings of TLE versus HC white matter connectome abnormalities. The white matter tracts are depicted by cross‐sectional areas (CSA) in both directionalities. When comparing TLE versus HC DWI connectomes measures, decreased CSA is observed across the whole‐brain in TLE patients, with the highest percentage of abnormal significant (corrected) connections in the subcortical‐to‐subcortical region, the frontal‐to‐frontal region, and the parietal‐to‐temporal region. CSA, cross‐sectional area of the white matter; DWI, diffusion‐weighted imaging; HC, healthy control; TLE, temporal lobe epilepsy.

The summary findings of both uncorrected and corrected percentages of significant connections or tracts (*p* ≤ .05) between the HC and TLE group are depicted in Figure 3 with results organized into five broader regions of the brain: frontal, occipital, parietal, temporal, and subcortical. The dominant directionality was revealed as decreased CSA in TLE patients when compared to that of HCs in both corrected and uncorrected results. Specifically, in the corrected results, TLE CSA was globally decreased across all brain regions when compared to HC CSA. The top significant connections of decreased CSA in TLE patients included subcortical‐to‐subcortical tracts, frontal‐to‐frontal tracts, and parietal‐to‐temporal tracts.

**FIGURE 4:**
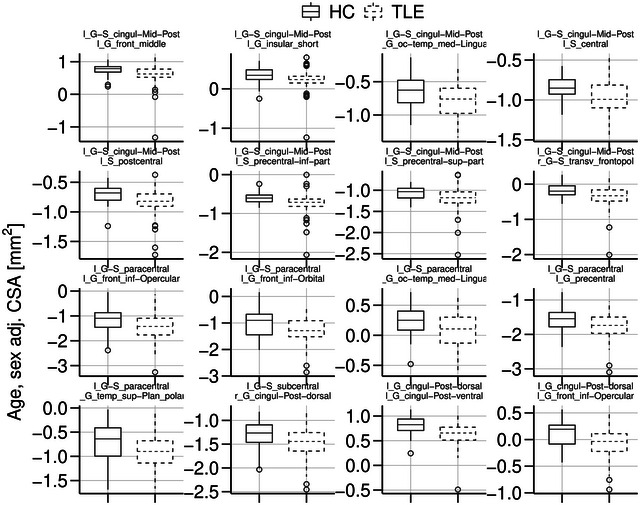
Representative TLE versus HC white matter connectome tract cross‐sectional areas. Illustrated here are 16 representative analyses after multiple comparisons correction of TLE versus HC DWI connectome abnormalities. In alignment with overall results, TLE patients exhibit lower white matter tract age and sex‐adjusted CSA when compared to HCs in more granular regions. The originating nodes and region locations are noted above each box plot. CSA, cross‐sectional area of the white matter; DWI, diffusion‐weighted imaging; HC, healthy control; TLE, temporal lobe epilepsy.

A panel of 16 representative tract connections with significantly (corrected) decreased CSA between TLE patients and HCs are illustrated in Figure 4. The box plots in this figure describe age and sex‐adjusted CSA differences between groups with the specific edges of these tracts displayed at the top of each graph. Our results showcase consistent significant reductions in TLE CSA across these 16 significant tracts when compared to HC CSA.

### 3.3 FBTC versus non‐FBTC in TLE patients

**FIGURE 5:**
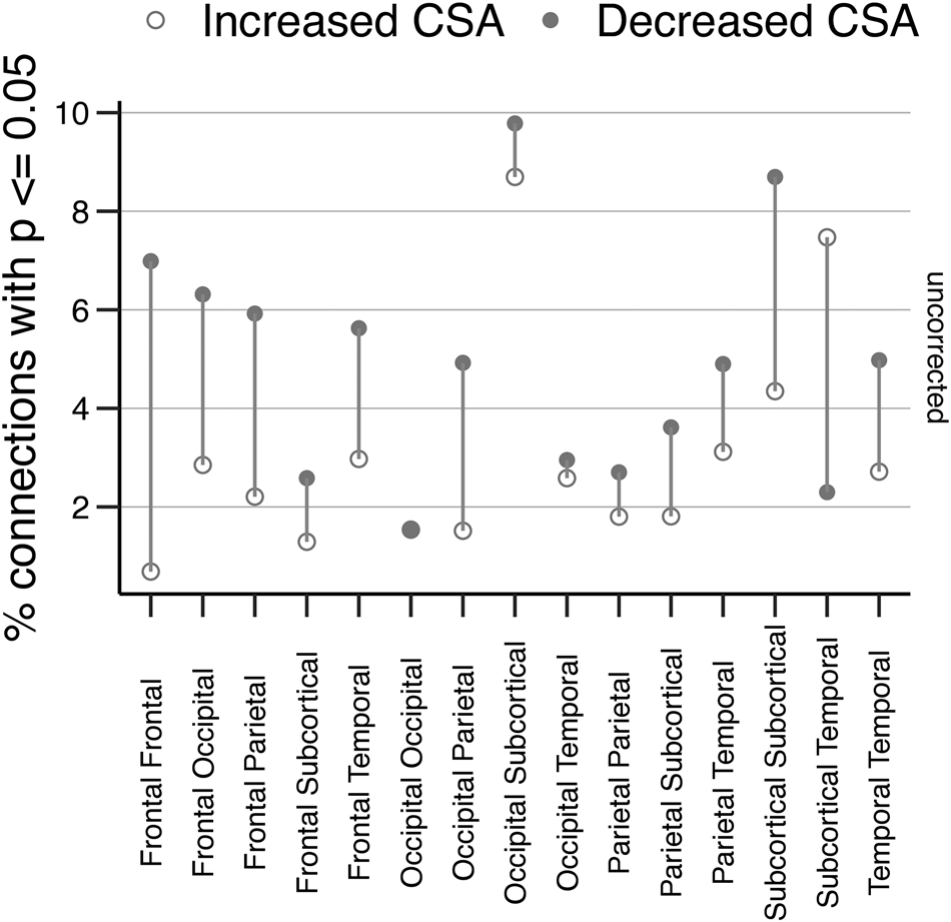
Summary findings of FBTC versus non‐FBTC TLE patients. The white matter tracts are depicted by cross‐sectional areas (CSA) in both directionalities. When comparing TLE patients with a positive history of FBTC seizures versus those who never had an FBTC seizure, uncorrected results illustrate global decreased white matter tract CSA in the FBTC group. The greatest percentages of tracts with uncorrected *p* ≤ .05 include the occipital‐to‐subcortical region, subcortical‐to‐subcortical region, and subcortical‐to‐temporal region. Corrected results result in no significant differences in the modest sample size and are not included in the figure. CSA, cross‐sectional area of the white matter; DWI, diffusion‐weighted imaging; FBTC, generalized tonic–clonic; HC, healthy control; TLE, temporal lobe epilepsy.

Next, we assessed the effect of FBTC seizures on CSA of the white matter connectome in TLE patients. The summary findings of the percentage of uncorrected significant tracts (*p* ≤ .05) when comparing TLE patients who had an FBTC seizure to TLE patients who have a negative FBTC seizure history are depicted in Figure 5. Our results indicate no significance after multiple comparisons correction; however, in the uncorrected results, the dominant contrast with the higher number of connections favors the direction where the FBTC group of TLE patients exhibited decreased CSA when compared to the non‐FBTC group. These results demonstrate modest and trend‐level findings of decreased CSA in the FBTC group of TLE patients compared to those who have never had an FBTC seizure.

### 3.4 Cognitive and neuropsychological considerations

Subject cognitive profiles and selected neuropsychological test scores were analyzed to correlate clinical variable signatures with DTI connectome data. Cognitive profiles were divided into three clusters: focal cognitive impairment, generalized cognitive impairment, and no cognitive impairment based on a previous study (Hermann et al., 2020).

**FIGURE 6:**
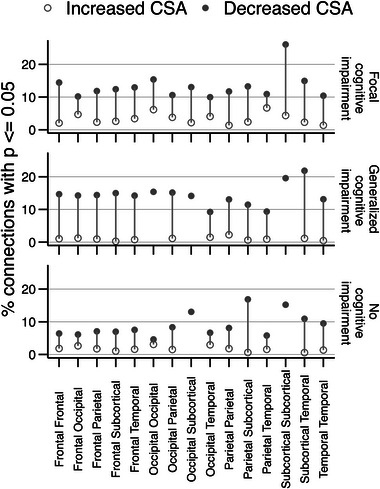
Summary of cognitive impairment clusters in temporal lobe epilepsy (TLE) patients with white matter connectome findings. The white matter connectome abnormalities of cognitive profiles were scrutinized in TLE versus cognitively intact healthy controls (HCs). Although these results did not survive correction, our uncorrected results illustrate modest and trend‐level findings in all three cognitive statuses in TLE patients versus HC with decreased white matter tract cross‐sectional area (CSA) as the dominant directionality. The clusters with cognitive impairments (both focal and generalized) highlight a greater percentage of uncorrected *p* ≤ .05 abnormal white matter tract connections with decreased CSA compared to the cluster with no cognitive impairment. The highest percentages of tracts represented are in the subcortical‐to‐subcortical region, the subcortical‐to‐temporal region, and the occipital‐to‐occipital region in the cognitively impaired profiles.

Our summary results of the uncorrected correlations of cognitive impairment and neuropsychological scores with the white matter connectome findings are illustrated in Figure 6. The focal cognitive impairment and generalized cognitive impairment TLE patients yielded similar results, exhibiting modest and trend‐level findings of greater than 100 connections, all of which exhibited decreased CSA when compared to HCs. The highest percentages of white matter tract correlations are found in the subcortical‐to‐subcortical, subcortical‐to‐temporal, and occipital‐to‐occipital regions. In TLE patients with no cognitive impairment, a smaller number of connections were revealed for decreased CSA when compared to HCs. In general, although all three cognitive statuses yielded decreased CSA in the TLE group compared to HCs, the cognitively impaired TLE groups demonstrated a modest trend with a greater proportion of significant tract differences in the decreased CSA directionality compared to those who are not cognitively impaired. These clusters are further detailed in Figures S2–S4 showing representative TLE with focal, generalized, and no cognitive impairment versus HC, respectively.

## 4 DISCUSSION

The present study investigates the structural DWI‐based differences in connectivity networks in the white matter of TLE patients and HCs using graph theory metrics. We further examined whether these differences were associated with a history of FBTC seizures and cognitive impairment status.

Our study is one of the first few large‐scale TLE DWI whole‐brain connectome studies in characterizing white matter network connectivity abnormalities, and the ECP dataset is the first to provide connectome quality multi‐shell DWI data with reverse phase encoded pairs of acquisitions. The current investigation expands on previous work from our lab on the ECP DWI dataset that has revealed the association of patient neighborhood deprivation with white matter connectivity abnormalities (Chu et al., 2023) as well as showcased the utility of graph theory metric in predicting group membership, described intrinsic structural TLE phenotype differences, and associated results with cognitive and clinical measures via support vector machine analyses (Garcia‐Ramos et al., 2023). Other previous literature exploring the TLE white matter connectome has revealed remodeling of connectome topology and structurally governed functional dynamics in 44 patients (Bernhardt et al., 2019) and comparisons of connectome fingerprint to postsurgical seizure outcomes in 52 patients (Morgan et al., 2022), and betweenness centrality analysis predicted postsurgical outcomes from presurgical evaluations in 47 patients (Gleichgerrcht et al., 2020).

### 4.1 Global connectome white matter networks of TLE versus HC

Our study employs TFNBS, which is a direct extension of the threshold‐free cluster enhancement used in tract‐based spatial statistics for correcting family‐wise errors in multiple comparisons. The extension is based on the fact that for networks, the neighborhood is determined by adjacency matrices and not the regular spatial 3D lattice. This gives unique advantages in that spatially nonlocal connections in the brain are considered when discerning statistically significant patterns. TFNBS is also unique when compared to the extensively used graph theoretic measures such as modularity, betweenness‐centrality, and so on. TFNBS provides family‐wise error‐corrected statistics at each connection level (i.e., it can provide statistical significance using the local network properties), whereas the graph theoretic measures provide statistics of the global network properties. The local approach offered by TFNBS is complementary to the global approach offered by graph theoretic measures and offers spatially specific biological interpretations that are not derivable using global approaches.

Through global analysis of the white matter connectome differences between TLE patients and HCs, our results reveal 97 significant connections (after statistical correction) highlighting widespread reductions in TLE CSA, with the largest proportion of significant tracts belonging to the subcortical‐to‐subcortical tracts, frontal‐to‐frontal tracts, and parietal‐to‐temporal tracts. These results make evident that TLE is linked with reductions in white matter connectivity networks, especially in the frontal and temporal regions of the brain. Recent studies have emphasized the importance of using whole‐brain structural connectomes for understanding TLE and predicting seizure outcomes (Bernhardt et al., 2015; Bonilha et al., 2015; Taylor et al., 2018). In turn, our study investigated graph theoretic measures of ms‐dMRI. Our results revealed widespread and global decreased CSA in TLE compared to HCs and provide evidence for its potential as a whole‐brain neuroimaging biomarker to identify and characterize TLE.

### 4.2 The effect of FBTC history

Patients with FBTC seizures are more prone to cognitive impairment, premature mortality, and psychiatric disorders and are at risk of several other disorders (Kodankandath et al., 2023). These seizures may be severe and rapid and involve the bilateral cortical, subcortical, and brainstem networks in the brain. It is surprising that there is very limited literature on structural connectome associations with FBTC seizures. Therefore, we assessed the impact of FBTC seizures on the white matter connectome and observed how the white matter network is affected in TLE patients with a history of FBTC seizures compared to TLE patients who never had an FBTC seizure. Our results indicate modest and trend‐level differences in uncorrected significant tracts. In these uncorrected results, the FBTC group had decreased CSA when compared to the non‐FBTC group and the lobes with the largest proportion of these tracts originated from occipital‐to‐subcortical, subcortical‐to‐subcortical, and subcortical‐to‐temporal tracts. Although these differences did not survive multiple comparisons correction, the uncorrected results were in the expected direction and were reported here both for transparency and to encourage and provide foundation for future studies to investigate further with a larger sample of patients with FBTC seizures.

### 4.3 Cognitive impairment status

Our study indicates that focal and generalized cognitive impairment showed very similar modest and trend‐level associations with white matter connectome findings when compared to HCs. In comparison to the group with no cognitive impairment, both cognitively impaired groups have a much greater proportion of tracts with decreased CSA. Interestingly, the number of significant connections is very similar between the focal and the generalized cognitively impaired groups. All groups had a greater number of significant tracts in the direction where CSA is decreased when compared to the controls. A larger sample of cognitive impaired TLE patients can further clarify and elaborate upon these findings.

Existing literature on DTI‐based white matter connectomics of TLE patients have highlighted abnormalities in language and memory. First, Kaestner et al. (2020) demonstrated that the structural connectome model was superior to models based on white matter microstructural or clinical features in predicting language impairment. Similarly, Munsell et al. (2019) showed that Boston Naming Test scores, a proxy for language performance, could be predicted using a model based on combined eigenvector node centrality. Network‐based superficial white matter analyses have shown abnormalities in graph theory metrics, such as decreased global efficiency, in language and memory‐impaired subjects (Balachandra et al., 2020). Structural network topology revealed increased characteristic path length in a subgroup of TLE patients with the most pronounced cognitive impairments across multiple domains, including memory and verbal comprehension (Hatton et al., 2020). Our results using the TFNBS analytic approach provide promising evidence for characterization of the white matter connectome as a useful and potential biomarker underlying cognitive impairment in TLE.

## AUTHOR CONTRIBUTIONS

**Daniel Y Chu**: Writing—original draft; methodology; validation; writing—review and editing; formal analysis; supervision. **Theodore P Imhoff‐Smith**: Writing—review and editing; formal analysis; validation; visualization; methodology. **Veena A Nair**: Investigation; conceptualization; data curation; project administration. **Timothy Choi**: Writing—review and editing; supervision. **Anusha Adluru**: Writing—review and editing; methodology; software; supervision; formal analysis. **Camille Garcia‐Ramos** and **Kevin Dabbs**: Writing—review and editing; visualization; methodology; software. **Jedidiah Mathis**: Writing—review and editing; methodology; conceptualization; investigation. **Andrew S Nencka**: Conceptualization; investigation; methodology; writing—review and editing. **Lisa Conant**: Conceptualization; writing—review and editing; methodology; resources; project administration. **Jeffrey R Binder**: Data curation; project administration; supervision; resources; conceptualization; investigation; funding acquisition. **Mary E Meyerand**: Conceptualization; investigation; funding acquisition; project administration; resources; supervision; data curation. **Andrew L Alexander**: Conceptualization; investigation; funding acquisition; writing—review and editing; project administration; supervision; resources. **Aaron F Struck**: Writing—review and editing; methodology; software; formal analysis; project administration; supervision; data curation; resources; investigation. **Bruce Hermann**: Methodology; validation; visualization; writing—review and editing; software; resources; supervision; data curation; conceptualization; investigation; project administration. **Vivek Prabhakaran**: Conceptualization; investigation; funding acquisition; methodology; data curation; supervision; resources; project administration; writing—review and editing. **Nagesh Adluru**: Conceptualization; investigation; writing—review and editing; writing—original draft; methodology; validation; visualization; software; formal analysis; project administration; resources; supervision; data curation; funding acquisition.

## FUNDING INFORMATION

None.

### PEER REVIEW

The peer review history for this article is available at https://publons.com/publon/10.1002/brb3.3643.

## Supporting information

Figure S1 Pre‐ and post‐data harmonization. The distributions of the log‐transformed edge weights (fiber bundle capacities) before and after the software update (from DV25 to DV26). We can notice that the data harmonization aligns the two distributions to a common distribution as evidenced by the aligned quantiles.

Figure S2 Representative TLE with focal cognitive impairment versus HC white matter connectome tract cross‐sectional areas. Illustrated here are 16 representative analyses after multiple comparisons correction of TLE with focal cognitive impairment versus HC DWI connectome abnormalities. The results indicate that TLE patients with focal cognitive impairment exhibit lower white matter tract age and sex‐adjusted expected CSA when compared to HCs. The originating nodes and region locations are noted above each box plot. CSA, cross‐sectional area of the white matter; DWI, diffusion‐weighted imaging; HC, healthy control; TLE, temporal lobe epilepsy.

Figure S3 Representative TLE with generalized cognitive impairment versus HC white matter connectome tract cross‐sectional areas. Illustrated here are 16 representative analyses after multiple comparisons correction of TLE with generalized cognitive impairment versus HC DWI connectome abnormalities. The results indicate that TLE patients with generalized cognitive impairment exhibit lower white matter tract age and sex‐adjusted expected CSA when compared to HCs. The originating nodes and region locations are noted above each box plot. CSA, cross‐sectional area of the white matter; DWI, diffusion‐weighted imaging; HC, healthy control; TLE, temporal lobe epilepsy.

Figure S4 Representative TLE with no cognitive improvement versus HC white matter connectome tract cross‐sectional areas. Illustrated here are 16 representative analyses after multiple comparisons correction of TLE who are cognitively intact versus HC DWI connectome abnormalities. The results indicate that TLE patients with no cognitive impairment exhibit lower white matter tract age and sex‐adjusted expected CSA when compared to HCs. The originating nodes and region locations are noted above each box plot. CSA, cross‐sectional area of the white matter; DWI, diffusion‐weighted imaging; HC, healthy control; TLE, temporal lobe epilepsy.

## Data Availability

A large‐scale dataset from the ECP was utilized for this study. Datasets from the ECP will be released via the Connectome Coordination Facility (CCF) established at Washington University in St. Louis. The CCF will become the long‐term repository for the de‐identified data and release at the earliest feasible time. Preprocessed DWI data are available from the team upon reasonable request following institutional guidelines. Associated scripts for data processing and analysis will be posted to GitHub (https://github.com/ecp‐dwi/brain_behavior). Readers can reach out to senior author Nagesh Adluru for assistance with using the scripts.
