# Supplementary figures and images for: Characterizing white matter connectome abnormalities in patients with temporal lobe epilepsy using threshold‐free network‐based statistics
Source: Brain Behav. 2024 Aug 5;14(8):e3643. doi: 10.1002/brb3.3643 (PMC11298711; doi:10.1002/brb3.3643)

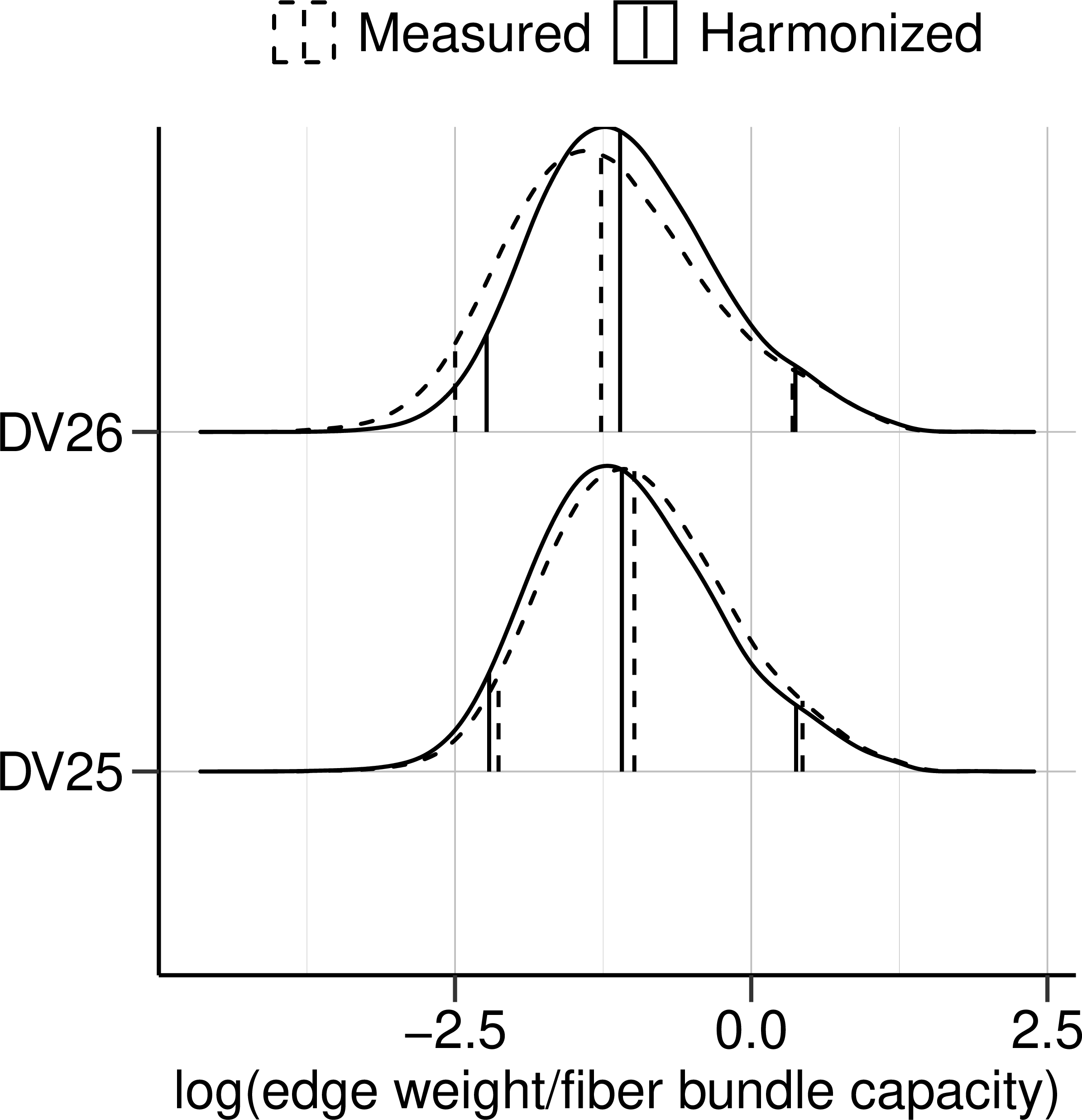

Supplement: Supplementary file 1 — Figure S1 Pre‐ and post‐data harmonization. The distributions of the log‐transformed edge weights (fiber bundle capacities) before and after the software update (from DV25 to DV26). We can notice that the data harmonization aligns the two distributions to a common distribution as evidenced by the aligned quantiles. [file BRB3-14-e3643-s003.png]

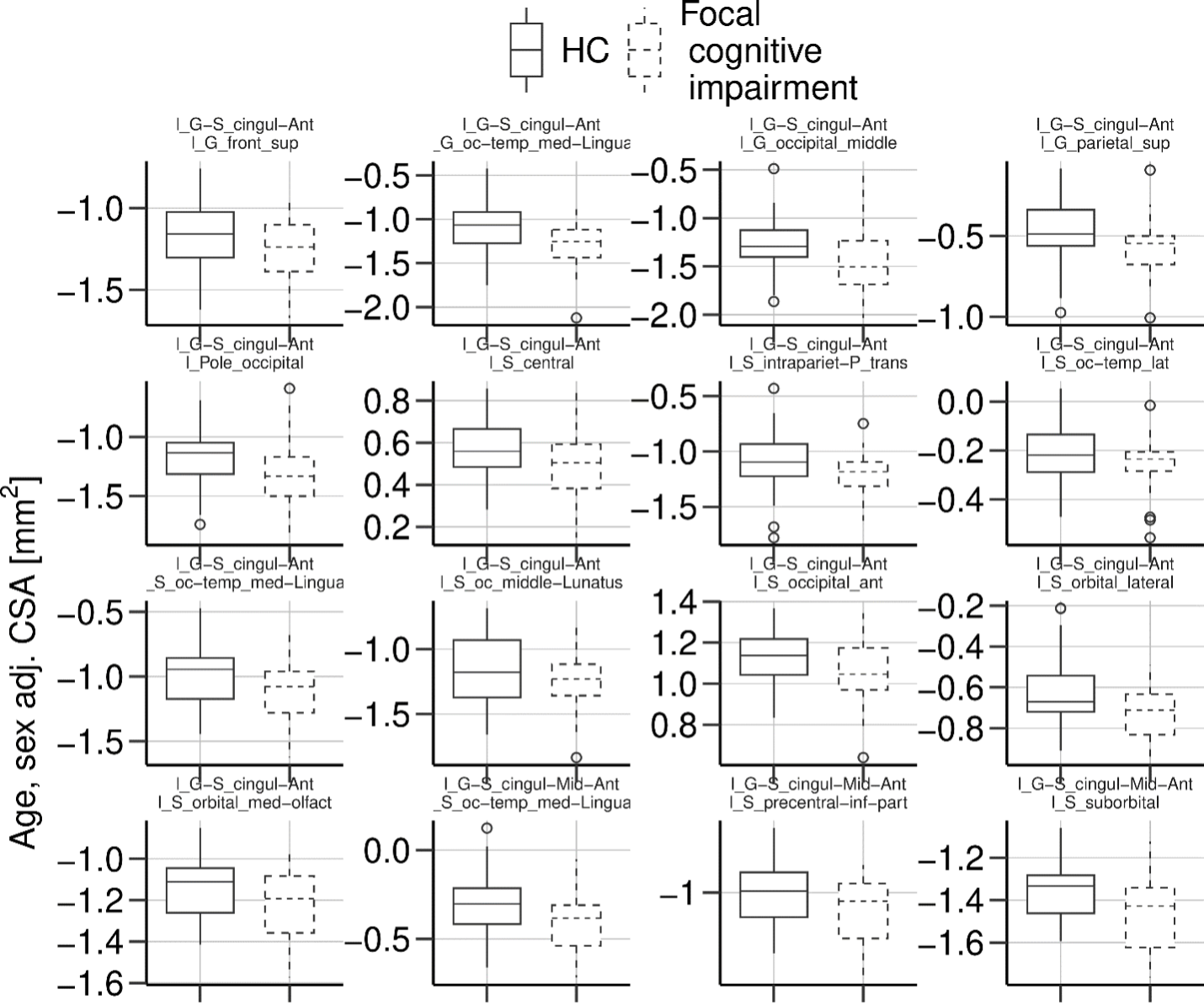

Supplement: Supplementary file 2 — Figure S2 Representative TLE with focal cognitive impairment versus HC white matter connectome tract cross‐sectional areas. Illustrated here are 16 representative analyses after multiple comparisons correction of TLE with focal cognitive impairment versus HC DWI connectome abnormalities. The results indicate that TLE patients with focal cognitive impairment exhibit lower white matter tract age and sex‐adjusted expected CSA when compared to HCs. The originating nodes and region locations are noted above each box plot. CSA, cross‐sectional area of the white matter; DWI, diffusion‐weighted imaging; HC, healthy control; TLE, temporal lobe epilepsy. [file BRB3-14-e3643-s002.png]

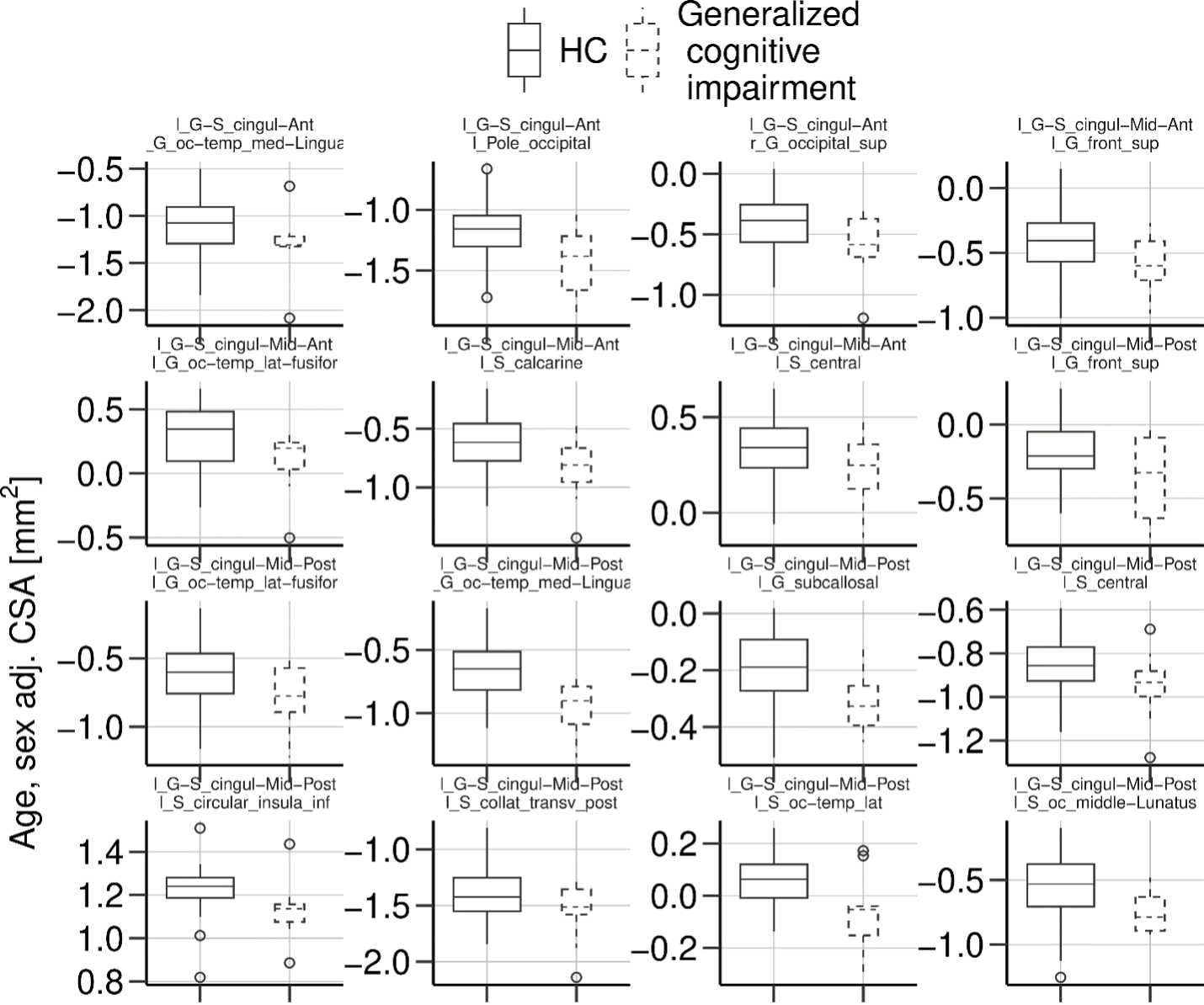

Supplement: Supplementary file 3 — Figure S3 Representative TLE with generalized cognitive impairment versus HC white matter connectome tract cross‐sectional areas. Illustrated here are 16 representative analyses after multiple comparisons correction of TLE with generalized cognitive impairment versus HC DWI connectome abnormalities. The results indicate that TLE patients with generalized cognitive impairment exhibit lower white matter tract age and sex‐adjusted expected CSA when compared to HCs. The originating nodes and region locations are noted above each box plot. CSA, cross‐sectional area of the white matter; DWI, diffusion‐weighted imaging; HC, healthy control; TLE, temporal lobe epilepsy. [file BRB3-14-e3643-s001.png]

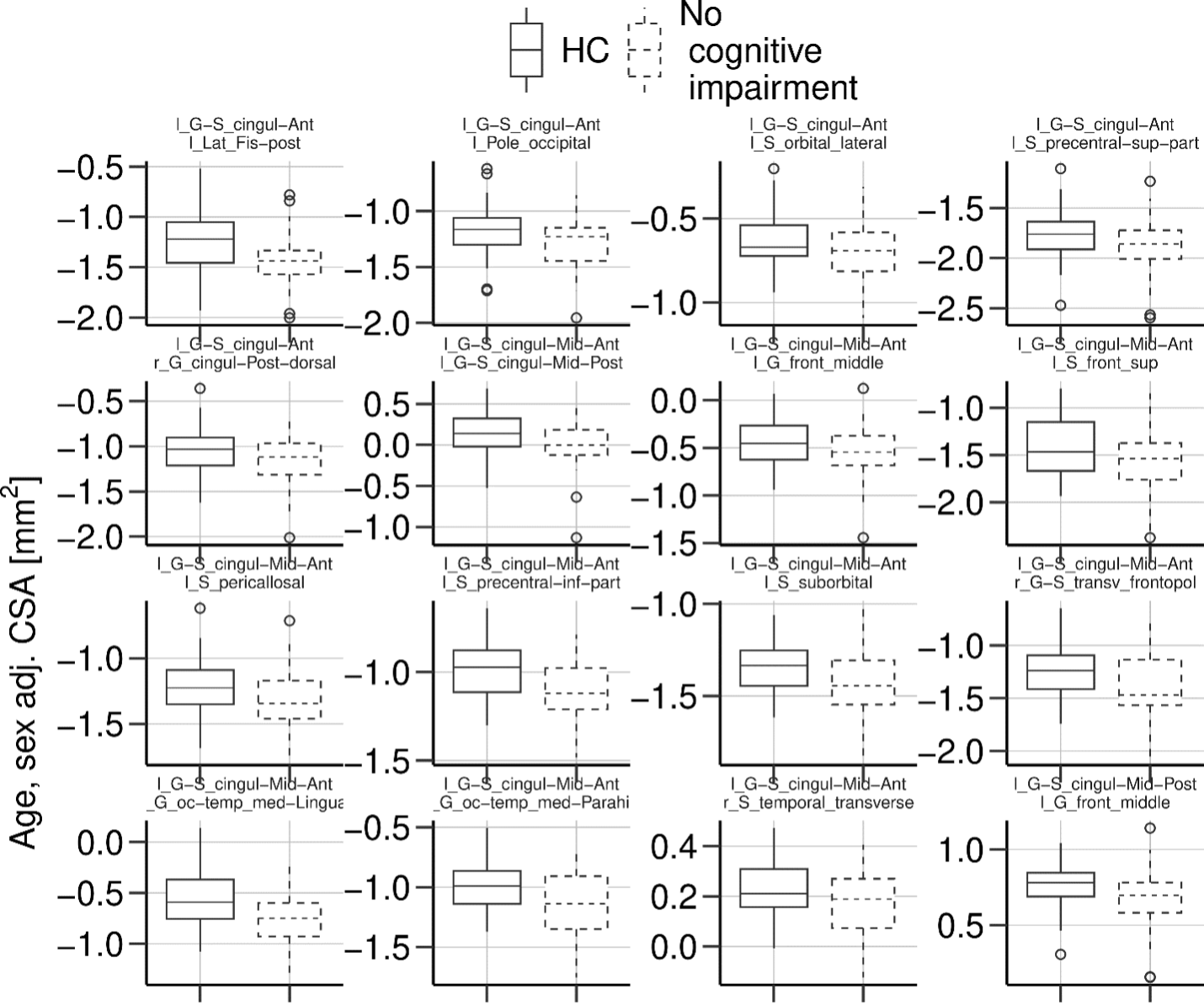

Supplement: Supplementary file 4 — Figure S4 Representative TLE with no cognitive improvement versus HC white matter connectome tract cross‐sectional areas. Illustrated here are 16 representative analyses after multiple comparisons correction of TLE who are cognitively intact versus HC DWI connectome abnormalities. The results indicate that TLE patients with no cognitive impairment exhibit lower white matter tract age and sex‐adjusted expected CSA when compared to HCs. The originating nodes and region locations are noted above each box plot. CSA, cross‐sectional area of the white matter; DWI, diffusion‐weighted imaging; HC, healthy control; TLE, temporal lobe epilepsy. [file BRB3-14-e3643-s004.png]
